# Coevolution of Lexical Meaning and Pragmatic Use

**DOI:** 10.1111/cogs.12681

**Published:** 2018-10-07

**Authors:** Thomas Brochhagen, Michael Franke, Robert van Rooij

**Affiliations:** ^1^ Institute for Logic Language and Computation, University of Amsterdam; ^2^ Department of Linguistics University of Tübingen; ^3^ Institute for Cognitive Science University of Osnabrück

**Keywords:** Semantics, Pragmatics, Language evolution, Evolutionary game theory

## Abstract

According to standard linguistic theory, the meaning of an utterance is the product of conventional semantic meaning and general pragmatic rules on language use. We investigate how such a division of labor between semantics and pragmatics could evolve under general processes of selection and learning. We present a game‐theoretic model of the competition between types of language users, each endowed with certain lexical representations and a particular pragmatic disposition to act on them. Our model traces two evolutionary forces and their interaction: (i) pressure toward communicative efficiency and (ii) transmission perturbations during the acquisition of linguistic knowledge. We illustrate the model based on a case study on scalar implicatures, which suggests that the relationship between underspecified semantics and pragmatic inference is one of coevolution.

## 1 Introduction

What is conveyed usually goes beyond what is said. A request for a blanket can be politely veiled by uttering “I’m cold”; a temporal succession of events can be communicated by the order in which conjuncts appear as in “I traveled to Paris and got married”; an invitation can be declined by saying “I have to work”. An influential explanation of the relation between the literal meaning of expressions and what they may convey in context is due to Grice (1975), who characterizes pragmatic use and interpretation as a process of mutual reasoning about rational language use. For instance, under the assumption that the speaker is cooperative and relevant, “I have to work” may be interpreted as providing a reason why the speaker will not be able to accept an invitation, going beyond its literal meaning. Some of these enrichments are rather ad hoc. Others show striking regularities, such as the use of ability questions for polite requests (“Could you please …?”), or certain enrichments of lexical meanings such as *and* to convey *and then*.

A particularly productive and well‐studied class of systematic pragmatic enrichments is scalar implicatures (Geurts, 2010; Hirschberg, 1985; Horn, 1984, Levinson, 1983). Usually, the utterance of a sentence like “I own some of Johnny Cash’s albums” will be taken to mean that the speaker does not own all of them. This is because, if the speaker instead owned them all, she could have used the word *all* instead of *some* in her utterance, thereby making a more informative statement. Scalar implicatures, especially the inference from *some* to *some but not all*, have been studied extensively, both theoretically (e.g., Chierchia, Fox, & Spector, 2012; van Rooij & de Jager, 2012; Sauerland, 2004) as well as experimentally (e.g., Bott & Noveck, 2004; Degen & Tanenhaus, 2015; Grodner, Klein, Carbary, & Tanenhaus, 2010; Goodman & Stuhlmüller, 2013; Huang & Snedeker, 2009). This makes them particularly suitable candidates for the study of the evolution of regular pragmatic inferences. While there is much dispute in this domain about many details, a position endorsed by a clear majority is that a scalar item like *some* is underspecified to mean *some and maybe all* and that the enrichment to *some but not all* is part of some regular process with roots in pragmatics. If this majority view is correct, the question arises how such a division of labor between semantics and pragmatics could have evolved.

Models of language evolution abound. There are simulation‐based models studying populations of communicating agents (Baronchelli, Puglisi & Loreto, 2008; Hurford, 1989; Lenaerts, Jansen, Tuyls & Vylder, 2005; Spike, Stadler, Kirby & Smith, 2016; Steels, 1995; Steels, 2011; Steels & Belpaeme, 2005) and there are mathematical models of language evolution, many coming from game theory (Blume, Kim, & Sobel, 1993; Huttegger, 2007; Nowak, 2006; Nowak & Krakauer, 1999; Skyrms, 2010; Wärneryd, 1993). Much of this work has focused on explaining basic properties such as compositionality and combinatoriality (e.g., Batali, 1998; Franke, 2016; Gong, 2007; Kirby, 2002; Kirby & Hurford, 2002; Kirby, Tamariz, Cornish & Smith, 2015; Nowak & Krakauer, 1999, Nowak, Plotkin & Jansen, 2000; Smith, Kirby & Brighton, 2003; Verhoef, Kirby & de Boer, 2014), but little attention has been paid to the interaction between conventional meaning and pragmatic use. What is more, many mathematical models explain evolved meaning as a regularity in the overt behavior of agents, abstracting from complex interactions between semantic representations and pragmatic use.

In contrast, we will here explicitly model language users’ representations of lexical meanings and their own particular manner of production and interpretation based on these lexical representations. Different types of pragmatic behavior—ways of deploying meaning in interaction—are represented using probabilistic models of pragmatic language use (Frank & Goodman, 2012; Franke & Jäger, 2016; Goodman & Frank, 2016). Replication and selection are described by the *replicator mutator dynamic*, a general and established model of evolutionary change in large and homogeneous populations (Hofbauer, 1985; Hofbauer & Sigmund, 2003; Nowak, 2006; Nowak et al., 2000; Nowak, Komarova & Niyogi, 2001). This approach allows us to study the interaction between (i) pressure toward communicative efficiency and (ii) infidelity in the transmission of linguistic knowledge, caused by factors such as inductive learning biases and sparse learning data (Kirby, Griffiths & Smith, 2014; Kirby & Hurford, 2002; Smith et al., 2003).

It is important to consider the effects of transmission of linguistic knowledge through (iterated) learning because neither semantic meaning nor pragmatic usage patterns are directly observable. Instead, language learners have to infer these unobservables from the observable behavior in which they result. We formalize this process as a form of Bayesian inference. Our approach thereby contains a well‐understood model of iterated Bayesian learning (Griffiths & Kalish, 2005, 2007) but combines it with functional selection, here formalized as the most versatile dynamic from evolutionary game theory, the replicator dynamic (Taylor & Jonker, 1978). Section 2 introduces this model.

Section 3 applies this model to a case study on scalar implicatures. It shows that inductive learning biases of Bayesian learners that favor simpler lexical meanings may play a role in preventing the lexicalization of scalar inferences and thereby lead to the emergence of Gricean‐like pragmatic reasoning types, but only if there is selective pressure for communicative efficiency and learnability. The results of this study are critically assessed in the light of the simplifying assumptions inherent in our abstract model in Section 4.

## 2 A model of evolving lexical representations and pragmatic behavior

### 2.1 Communicative success and learnability

The emergence and change in linguistic structure are influenced by many factors in complex ways (Benz, Jäger, & van Rooij, 2005; Steels, 2011; Tamariz & Kirby, 2016). Social and ecological pressures determine communicative needs, while biology determines the architecture that enables and constrains the means by which they can be fulfilled. In the following sections, our focus lies on cultural aspects, wherein processes of linguistic change are viewed as shaped by language use and its transmission, that is, as a result of a process of cultural evolution (Pagel, 2009, Thompson, Kirby, & Smith, 2016).

The idea that language is an adaptation to serve a communicative function is fundamental to many synchronic and diachronic analyses, at least since Zipf’s (1949) explanation of word frequency rankings as a result of competing hearer and speaker preferences (e.g., Horn, 1984; Jäger & van Rooij, 2007; Jäger, 2007a; Kirby et al., 2015; Piantadosi, 2014; Martinet, 1962). If processes of selection, such as conditional imitation or reinforcement, favor communicative efficiency, languages are driven toward semantic expressivity (e.g., Nowak & Krakauer, 1999; Skyrms, 2010). But pressure toward communicative efficiency is not the only force that shapes language. Learnability is another. Natural languages need to be learnable to survive their faithful transmission across generations. Furthermore, the effects of even small learning biases can have striking effects on an evolving language in a process of iterated learning (Kirby & Hurford, 2002; Smith et al., 2003; Kirby et al., 2014).

While natural languages are pressured for both communicative efficiency and learnability, these forces may pull in opposite directions (Christiansen & Chater, 2008:x7). Their opposition becomes particularly clear when considering the extreme (Kemp & Regier, 2012; Kirby et al., 2015). A language consisting of a single form‐meaning association is easy to learn but likely bad for communication with peers. Conversely, a language that lexicalizes distinct forms for a large number of different meanings may be better for communication but more challenging to acquire.

### 2.2 The replicator mutator dynamic

An elegant formal approach to capture the interaction between selection for communicative efficiency and learnability is the *replicator mutator dynamic* (Hofbauer, 1985; Hofbauer & Sigmund, 2003; Nowak, 2006; Nowak et al., 2001, 2000). The RMD is a population dynamic which describes the average change in the composition of a community of agents of different types. The dynamic assumes a virtually infinite population, so that it is able to abstract away from what individual agents do. It describes the most probable trajectory of change in the population vector $\vec{x}$, in which each component $x_{i}$ gives the relative frequency of type *i* and $\sum_{i}x_{i}\;=\;1$. In its simplest discrete‐time formulation, the RMD defines the frequency $x_{i}^{\prime }$ of each type *i* in the population at the next time step as a function of (i) the frequency $x_{i}$ of each type *i* before the update step, (ii) the fitness $f_{i}$ of each type *i* before the update, and (iii) the probability $Q_{\mathrm{ji}}$ that an agent who observes the behavior of an agent with type *j* ends up acquiring type *i* (with $\sum_{k}Q_{\mathrm{jk}}\;=\;1$ for all *j*):

(1) $$x_{i}^{\prime }=\sum_{j}\frac{x_{j}f_{j}Q_{\mathrm{ji}}}{\Phi },$$

where $\Phi \;=\;\sum_{k}x_{k}f_{k}$ is the average fitness in the population.

The RMD consists of two components: fitness‐based selection and transmission biases encoded in the so‐called mutation matrix *Q*. This becomes most transparent when we consider an equivalent formulation in terms of a step‐wise application of the discrete‐time replicator dynamic (Taylor & Jonker, 1978) on the initial population vector $\vec{x}$ and a subsequent multiplication with the mutation matrix *Q*:

(2) $$x_{i}^{\prime }\;=\;{\left(M\left(\text{RD}\left(\vec{x}\right)\right)\right)}_{i},$$

where

$${\left(\text{RD}(\vec{x})\right)}_{i}=\frac{x_{i}f_{i}}{\Phi }\;\text{and}\;{\left(M\left(\vec{x}\right)\right)}_{i}={\left(\vec{x}\cdot Q\right)}_{i}={\left(\sum_{j}x_{j}Q_{\mathrm{ji}}\right)}_{i}.$$

If there is no mutation, so that types are always replicated faithfully with $Q_{\mathrm{ji}}\;=\;1$ whenever *j* = *i*, the RMD reduces to the replicator dynamic. If fitness is the same for all types, $f_{i}\;=\;f_{j}$ for all *i* and *j*, the RMD reduces to a process of iterated mutation. Appendix A provides an abstract minimal example.

### 2.3 Interpreting the replicator mutator dynamic

The RMD is an abstract description of population‐level changes. It abstracts away, to the extent possible, from specific assumptions about how agents interact and what exactly causes selection and mutation. To understand this better, the following looks at each component in isolation: first replication, then mutation.

According to the replicator dynamic, the relative frequency $x_{i}$ of type *i* increases proportional to its average fitness in the population. This dynamic is popular and versatile because it can be derived from many processes of biological and cultural transmission and selection (for overview and several derivations, see Sandholm, 2010). Under a biological interpretation, the model describes population‐level changes when the number of offsprings under single‐parent reproduction is proportional to the parent’s fitness. Under a cultural interpretation, examples of concrete agent‐level processes that lead to population‐level changes as described by the replicator dynamic include conditional imitation (e.g., Helbing, 1996; Schlag, 1998) or reinforcement learning (e.g., Beggs, 2005; Börgers & Sarin, 1997). In conditional imitation, for instance, each agent carries a fixed type *i* which it may change to a type *j* when it happens to observe another agent using type *j*. Imitation occurs with a probability that is a monotonic function of $f_{j}$, that is, of how well *j* fares against the population: the better the behavior *j*, the more likely it is to be imitated.^1^

If all types have equal fitness at all times, the replicator mutator dynamic reduces to a process of iterated mutation. Mutation can be interpreted as biological or cultural as well. Under a biological interpretation where types are genetically encoded behavioral traits, $Q_{\mathrm{ji}}$ is the probability by which offspring of type *j* genetically mutates into type *i*. Under a cultural interpretation where types are transmitted by observation or learning, such as in conditional imitation, $Q_{\mathrm{ji}}$ is the probability that an agent trying to learn, adopt, or imitate the behavior of an agent with type *j* acquires type *i*.

In the following application to language evolution, we prefer a cultural interpretation of the mutator component as the perturbation induced by how easy different linguistic traits are learnable from observation of language use. In this way, the RMD contains a chain of iterated (Bayesian) learning as a special case (Griffiths & Kalish, 2005, 2007). Nothing of current relevance hinges on whether the replicator component is interpreted as a biological process or a cultural process. We believe that this flexibility of interpretation is an asset. It abstracts away from specific assumptions and focuses on a general and versatile description of fitness‐based selection. Still, to have something concrete in mind when interpreting the following application, a derivation of the RMD from a specific scheme of agent‐level conditional imitation is given in the Supplementary Material.

### 2.4 Fitness and learnability of lexical meanings and pragmatic strategies

Our goal is to apply the RMD to the evolution of lexical representations and pragmatic behavior. This requires determining three things: (i) what the relevant types are, (ii) how fitness of a type derives from its communicative success, and (iii) how the mutation matrix *Q* is computed. These issues are addressed, one by one, in the following.

#### 2.4.1 Types: Lexica and pragmatic strategies

Types are what evolution promotes or demotes. Its type determines an agent’s behavior and thereby an agent’s fitness, so that it makes sense to speak of the fitness of a type itself. To study the joint evolution of semantic meaning and pragmatic use, types are pairs consisting of a lexicon and a linguistic strategy of language use.

Agents play signaling games, in which the speaker wants to communicate a world state *s* with a message *m* to a hearer who receives *m* but does not know *s* (e.g., Lewis, 1969; Skyrms, 2010). A lexicon associates each message with a set of states. A linguistic behavior specifies a probabilistic speaker rule (a probabilistic choice of message for each state) and a probabilistic hearer rule (a probabilistic choice of state for each message) given a lexicon.

Lexica codify the truth‐conditions of expressions. A convenient way to represent lexica is by (|*S*|,|*M*|)‐Boolean matrices, where *S* is a set of states (meanings) and *M* a set of messages (forms available in the language). For example, suppose that there are two relevant world states $S\;=\;\{s_{\exists \neg \forall },s_{\forall }\}$. In state $s_{\exists \neg \forall }$, Chris owns some but not all of Johnny Cash’s albums, while in $s_{\forall }$ Chris owns them all. Suppose that there are two messages $M\;=\;\{m_{\mathrm{some}},\;m_{\mathrm{all}}\}$ where $m_{\mathrm{some}}$ is short for a sentence like *Chris owns some of Johnny Cash’s albums* and $m_{\mathrm{all}}$ for the same sentence with *some* replaced by *all*. Lexica then assign a Boolean truth value, either 0 for false or 1 for true, to each state‐message pair. The following two lexica are minimal examples for the distinction between a lexicalized upper bound for *some*, in $L_{\mathrm{bound}}$, and the widely assumed logical semantics with only a lower bound, in $L_{\mathrm{lack}}$. 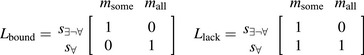


Linguistic strategies define dispositions to produce and interpret messages given a lexicon. We distinguish between two kinds strategies. *Literal interlocutors* produce and interpret messages literally, being guided only by their lexica. *Pragmatic interlocutors* instead engage in mutual reasoning to inform their choices. Recent game‐theoretic (e.g., Benz, 2006; Benz & van Rooij, 2007; Franke & Jäger, 2014; Jäger, 2007b) and probabilistic models of rational language use (e.g., Frank & Goodman, 2012; Franke & Jäger, 2016; Goodman & Frank, 2016) capture different types of pragmatic behavior in a reasoning hierarchy. In the following sections, we aim at a general formulation of speaker and listener behavior which is as simple and as practical as possible for our current purposes but still in line with both the game‐theoretic and the Bayesian traditions.

The hierarchy’s bottom, level 0, corresponds to literal language use, as in Eqs. (3) and (4). Pragmatic language users of level *n*+1 act (approximately) rational with respect to level‐*n* behavior of their interlocutors, as in Eqs. (5) and (6).

(3) $$H_{0}\left(s|m;L\right)\;\propto pr\left(s\right)L_{\left[s,m\right]}$$

(4) $$S_{0}\left(m|s;L\right)\;\propto \exp\left(\lambda \;L_{\left[s,m\right]}\right)$$

(5) $$H_{n+1}\left(s|m;L\right)\;\propto pr\left(s\right)S_{n}\left(m|s;L\right)$$

(6) $$S_{n+1}\left(m|s;L\right)\;\propto \exp\left(\lambda \;H_{n}\left(s|m;L\right)\right)$$

According to (3), a literal hearer’s interpretation of a message depends on her lexicon and her prior over states, *pr* ∈ Δ(*S*), which is in the following assumed flat for simplicity. Literal interpreters thereby choose an arbitrary true interpretation for each message according to their lexicon. Pragmatic hearers, defined in (5), instead use Bayes’ rule to weigh interpretations based on a conjecture about speaker behavior. Speaker behavior is regulated by a soft‐max parameter *λ* ≥ 0 (Luce, 1959; Sutton & Barto, 1998). As *λ* increases, choices approximate strict maximization of expected utilities. Expected utility of a message *m* in state *s* for a level *n* + 1 speaker is here defined as $H_{n}\left(s|m;L\right)$, the probability that the hearer will assign to or choose the correct meaning.^2^ For literal speakers, utility only tracks truthfulness. Literal speakers choose any true message with equal probability but may send false messages as well with a probability dependent on *λ*.

In words, an agent’s linguistic behavior—what message to send when or which message to interpret how—is defined by (i) her reasoning level and (ii) her lexicon. In what follows, this is what we identify an agent’s type with, in order to analyze under which conditions combinations of (i) and (ii) emerge through cultural evolution. Intuitively, literal behavior (level‐0 reasoning) results from unreflected language use. Such agents produce and comprehend solely based on the truth‐conditions of their lexicon. In a Gricean spirit, pragmatic behavior (level‐*n* + 1 reasoning) results from reasoning about rational language use. Such agents produce and comprehend by reasoning about how they themselves would interpret or use expressions. Higher order behavior is therefore, even if in tendency rational, quite simple. It does not assume agents to know their interlocutor’s type when interacting (e.g., what lexicon the interlocutor is using); they simply act based on a conjecture about language use derived from their own (solipsistic) perspective.

The following examples illustrate these behaviors numerically. A literal interpreter with lexicon $L_{\mathrm{bound}}$ assigns *s*∃¬∀ a probability of $H_{0}\left(s\exists \neg \forall |m_{\mathrm{some}};L_{\mathrm{bound}}\right)\;=\;1$ after hearing $m_{\mathrm{some}}$, while a literal interpreter with $L_{\mathrm{lack}}$ has $H_{0}\left(s\exists \neg \forall |m_{\mathrm{some}};L_{\mathrm{lack}}\right)\;=\;0.5$: 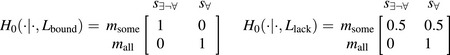 In contrast, pragmatic hearers of level 1 have the following interpretative behavior for *λ* = 1:
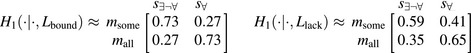
This is the outcome of reasoning about their level‐0 speaker counterparts with *λ* = 1:
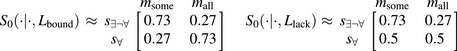
With low *λ*, speakers choose true messages with more slack. Reasoning over this behavior, therefore, also results in a weaker association of messages with not only true states in receivers but also in a slightly stronger association of *m*_some_ with *s*_∃¬∀_ over ∀ for *L*_lack_ users, because they reason that $S_{0}\left(m_{\mathrm{some}}|s_{\exists \neg \forall };L_{\mathrm{lack}}\right)\;>\;S_{0}\left(m_{\mathrm{some}}|s_{\forall };L_{\mathrm{lack}}\right)$. For *λ* = 20, there will be less slack in literal speaker behavior:
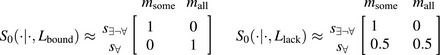
And accordingly less slack in level 1 pragmatic interpretation:
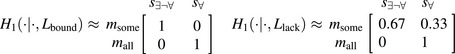
Lastly, turning to types that have no bearing on the choices of hearers of level 1, with *λ* = 1 pragmatic speakers of level 1 have:
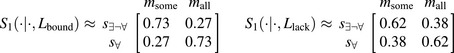
For *λ* = 20, pragmatic speaker behavior of level 1 is instead as follows:
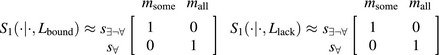


In contrast to their literal counterparts of level 0, pragmatic agents of level 1 who use *L*_lack_ associate *m*_some_ preferentially with *s*_∃¬∀_. This association is not perfect, and usually less strong than what agents with a lexicalized upper bound in *L*_bound_ can achieve—with or without pragmatic reasoning. Higher order reasoning beyond level 1 leads to stronger associations of *m*_some_ and *s*_∃¬∀_ also for the receiver. Still, the case study presented in Section 3 will consider sender and receiver behavior at levels 0 and 1, as the latter are the simplest pragmatic reasoning types which show a tendency to communicatively attuned pragmatic enrichment.

When it comes to competition between types of use of lexicon $L_{\mathrm{bound}}$, pragmatic reasoning at level 1 is not advantageous. The reason for this is that literal use of $L_{\mathrm{bound}}$ already endows agents with a behavioral strategy that associates a single state with a single message (in tendency; depending on *λ* for senders). For $L_{\mathrm{bound}}$‐receivers of level 1, reasoning over stochasticity introduced at $S_{0}\left(\cdot |\cdot ,L_{\mathrm{bound}}\right)$ will generally decrease the association of one state with one message. This decrease is only slight if *λ* is high, but nevertheless present. That is to say, level‐1 reasoning does not necessarily confer a functional advantage. For some types, such as users of $L_{\mathrm{bound}}$, literal signaling is preferable. In sum, the definition of types introduced here constitutes conditions that are purposefully averse to what we would like to show; the evolution of a division of labor between semantics and pragmatics is not immediate just based on fitness‐based selection, to which we turn next.

#### 2.4.2 Fitness and fitness‐based selection based on communicative success

In the context of language evolution, fitness is usually associated with the ability to successfully communicate with other language users from the same population (e.g., Nowak et al., 2000; Nowak, Komarova, & Niyogi, 2002; Nowak & Krakauer, 1999). Concretely, the fitness of type *i* is its average expected communicative success, or *expected utility* (EU), given the relative frequencies $x_{j}$ of types *j* in the current population:

$$f_{i}=\sum_{j}x_{j}\text{EU}\left(t_{i},t_{j}\right).$$

The expected utility $\text{EU}(t_{i},t_{j})$ for type *i* when communicating with type *j* is the average success of *i* when talking or listening to *j*. If, as standardly assumed, agents are speakers half of the time, this yields:

$$\text{EU}\left(t_{i},t_{j}\right)=1/2{\text{EU}}_{S}\left(t_{i},t_{j}\right)+1/2{\text{EU}}_{H}\left(t_{i},t_{j}\right),$$

where ${\text{EU}}_{S}\left(t_{i},t_{j}\right)$ and ${\text{EU}}_{H}\left(t_{i},t_{j}\right)$ are the expected utilities for *i* as a speaker and as a hearer when communicating with *j*, defined as follows, where $n_{i}$ and $n_{j}$ are type *i*’s and type *j*’s pragmatic reasoning types and $L_{i}$ and $L_{j}$ are their lexica:

$$\begin{array}{cc} & {\text{EU}}_{S}\left(t_{i},t_{j}\right)=\sum_{s}P\left(s\right)\sum_{m}S_{n_{i}}\left(m|s;L_{i}\right)\sum_{s^{\prime }}H_{n_{j}}\left(s^{\prime }|m;L_{j}\right)\delta \left(s,s^{\prime }\right),\\ & {\text{EU}}_{H}\left(t_{i},t_{j}\right)={\text{EU}}_{S}\left(t_{j},t_{i}\right). \end{array}$$

As usual, we assume that agents are cooperative: $\delta (s,s^{\prime })\;=\;1$ iff $s\;=\;s^{\prime }$ and 0 otherwise.

In words, expected utility $\text{EU}(t_{i},t_{j})$ quantifies how successful communication between agents of types *i* and *j* is, with each type’s behavior resulting from a combination of a lexicon and a reasoning level (Section 2.4.1). Fitness $f_{i}$ indicates how well type *i* fares in a population $\vec{x}$ where the probability of meeting a type *j* is $x_{j}$.

#### 2.4.3 Learnability

In biological evolution, where types are expressed genetically, transmission infidelity comes into the picture through infrequent and mostly random mutation and genetic drift (Kimura, 1983). However, an agent’s lexicon and a disposition for pragmatic reasoning are likely not inherited genetically. They need to be learned from observation, such as in conditional imitation (see Supplementary Material). Concretely, when agents learn from or imitate type *j*, they observe the overt linguistic behavior of type *j* and need to infer the covert type that most likely produced the observed behavior.

Iterated learning is a process in which languages are learned repeatedly from the observation of linguistic behavior of agents who have themselves acquired their behavior from observation and inference. In the simplest case there is a single teacher and a single learner in each generation (e.g., Brighton, 2002; Kirby, 2001). After sufficient training the learner becomes a teacher and produces behavior that serves as input for a new learner. Due to the pressure exerted toward learnability, iterated learning alone generally leads to simpler and more regular languages (see Kirby et al., 2014; Tamariz & Kirby, 2016 for recent surveys).

Following Griffiths and Kalish (2005, 2007), we model language acquisition as a process of Bayesian inference in which learners combine the likelihood of a type producing the witnessed learning input with prior inductive biases. In a Bayesian setting these biases can be codified in a prior *P* ∈ Δ(*T*), which reflects the amount of data a learner requires to faithfully acquire the language of the teacher (cf. Griffiths & Kalish, 2007:450).

The prior’s influence depends on particulars of the learning or inference process. Early simulation results suggested that weak biases could be magnified by exposing learners to only small data samples (e.g., in Brighton, 2002). Griffiths and Kalish (2007) proved that if learners adopt a random type as a sample from their posterior beliefs about which types may have generated the learning data that they saw, the population distribution of types will eventually come to match the learners’ prior distribution over types exactly. More deterministic strategies such as the adoption of the type with the highest posterior probability, so‐called *maximum a posterior estimation* (MAP), increase the influence of both the prior and the data (Griffiths & Kalish, 2007; Kirby, Dowman, & Griffiths, 2007). In the following, we use a parameter *l* ≥ 1 to modulate between posterior sampling and the MAP strategy. When *l* = 1, learners sample from the posterior. The learners’ propensity to maximize the posterior grows as *l* increases.^3^

Let *D* be the set of possible data that learners may be exposed to. This set contains all sequences of state‐message pairs of length *k*, for example, $\left\langle \left\langle s_{1},m_{1}\right\rangle ,\ldots ,\left\langle s_{k},m_{k}\right\rangle \right\rangle$. As *k* increases, learners have more data to base their inference on and so tend to recover the true types that generated a given sequence with higher probability. The mutation matrix *Q* of the replicator mutator dynamic in (1) can then be defined as follows: $Q_{\mathrm{ji}}$ is the probability that a learner acquires type *i* when learning from an agent of type *j*. The learner observes length‐*k* sequences *d* of state‐message pairs, but the probability $P(d|t_{j})$ with which sequence $d\;=\;\langle \left\langle s_{1},m_{1}\right\rangle ,\ldots ,\left\langle s_{k},m_{k}\right\rangle \rangle$ is observed depends on type *j*’s linguistic behavior:

$$P\left(d=\left\langle \left\langle s_{1},m_{1}\right\rangle ,\ldots ,\left\langle s_{k},m_{k}\right\rangle \right\rangle |t_{j}\right)=\prod_{i=1}^{k}S_{n_{j}}\left(m_{i}|s_{i};L_{j}\right),$$

where, as before, $n_{j}$ is *j*’s pragmatic reasoning level and $L_{j}$ is *j*’s lexicon. For a given observation *d*, the probability of acquiring type *i* is $F(t_{i}|d)$, so that:

$$Q_{\mathrm{ji}}=\sum_{d\in D}P\left(d|t_{j}\right)F\left(t_{i}|d\right).$$

The acquisition probability $F(t_{i}|d)$ given datum *d* is obtained by probability matching *l* = 1 or a tendency toward choosing the most likely type *l* > 1 from the posterior distribution *P*(·|*d*) over types given the data. This is calculated by Bayes’ rule:

$$\begin{array}{cc} & F\left(t_{i}|d\right)\propto P{\left(t_{i}|d\right)}^{l}\;\;\text{and}\;\\ & P\left(t_{i}|d\right)\propto P\left(t_{i}\right)P\left(d|t_{i}\right). \end{array}$$

When *l* = 1, learners sample a type to adopt from their posterior distribution which they obtain from observing the teacher produce utterances in particular states. From Griffiths and Kalish (2007), we know that under *l* = 1 iterated Bayesian learning will converge to a population proportion that exactly matches the agents’ prior over types. If *l* > 1 learners tend to pick types which are a posteriori more likely with a higher probability than less likely types. Even in the absence of a prior bias, iterated Bayesian learning with *l* > 1 can lead to populations in which a particular type $t_{i}$ has a higher proportion than other types. For *l* > 1, much hinges on particular asymmetries in production likelihoods $P(d|t_{j})$ and therefore on the composition of the set of types in general. Intuitively put if there are many types $t_{j}$ which all happen to (“erroneously”) produce data that are most likely produced (“correctly”) by $t_{i}$, this can lead to a larger proportion of $t_{i}$ in the long run even when priors over types are uniform. Appendix B provides an abstract example for these likelihood‐driven effects.

### 2.5 Model summary

Communicative success and learnability is central to the cultural evolution of language. These components can be modelled, respectively, as replication based on a measure of fitness in terms of communicative efficiency and iterated Bayesian learning. Their interaction is described by the discrete time replicator mutator dynamic in (1), repeated here:

$$x_{i}^{\prime }=\sum_{j}Q_{\mathrm{ji}}\frac{x_{j}f_{j}}{\sum_{h}x_{h}f_{h}}.$$

This equation defines the frequency $x_{i}^{\prime }$ of type *i* at the next time step, based on its frequency $x_{i}$ before the update step, its fitness $f_{i}$, and the probability that a learner infers *i* when observing the behavior of a type‐*j* agent. Fitness‐based replication can be thought of as biological (fitness as expected relative number of offspring) or cultural evolution (fitness as likelihood of being imitated or repeated; see Supplementary Material for a concrete example). A type’s communicative success depends on how well it communicates within its population while its learnability depends on the fidelity by which it is inferred by new generations of learners. The learners’ task is consequently to perform a joint inference over types of linguistic behavior and lexical meaning.

The model has three parameters: *λ* regulates the degree to which speakers choose messages that appear optimal from the point of view of the agent’s own utility measure (which may be unrelated to the expected utility when communicating with a given population); *k* is the length of observations received by each language learner; *l* regulates where the learners’ inference behavior lies on a spectrum from probability matching to acquisition of the most likely teacher type.

## 3 Case study: Scalar implicatures

This section addresses the question under which conditions the division of labor, which was described in Section 1, between underspecified semantics and pragmatic enrichment could have evolved. We consider what are perhaps two of the simplest nontrivial setups that speak to this matter and reflect on their limitations in Section 4. Section 3.1 first investigates a minimal example intended to isolate effects of replication and mutation and to gain insights into the sense in which semantic meaning and pragmatic use could be said to coevolve. We then turn to a larger and more realistic setup in Section 3.2. Section 3.3 describes simulations for this case and their results.

### 3.1 Coevolution of semantics and pragmatics in a restricted type space

**Figure 1:**
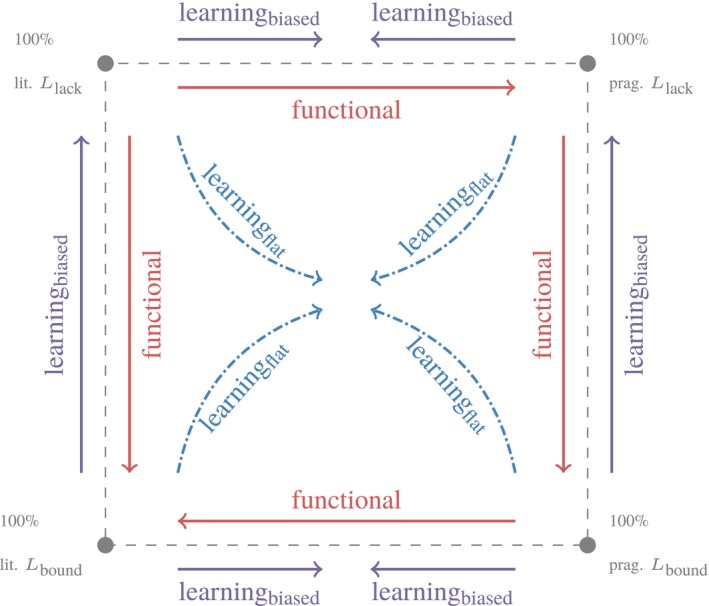
Sketch of dynamics on a two‐dimensional type space with four types. The *y*‐axis represents the proportion of types with $L_{\mathrm{lack}}$; the *x*‐axis represents that of pragmatic ones. Arrows sketch population trajectories under functional pressure (replicator steps, red arrows) and learnability pressure (mutator steps) with either a flat prior (blue dash‐dotted arrows) or a prior that favors $L_{\mathrm{lack}}$ (solid purple arrows).

This section illustrates how replication and mutation act on the evolution of semantic meaning and pragmatic use in the simplest nontrivial type space, which consists of only the four types from Section 2.4.1: a type has either lexicon $L_{\mathrm{lack}}$ or $L_{\mathrm{bound}}$ and it is either a literal (level‐0) or a pragmatic (level‐1) language user. We may think of such a population as a point in a two‐dimensional space. The grey dotted square in Fig. 1 outlines this space. The space’s first dimension determines the proportion of types that lexicalize ambiguous $m_{\mathrm{some}}$ (the *y*‐axis in Fig. 1). The second dimension determines the proportion of pragmatic language users (the *x*‐axis in Fig. 1). Populations at the corners of this space are monomorphic; all agents in them are of a single type. Points away from the corners represent mixed populations of various types.^4^ We can predict general trends in evolutionary trajectories in this space based on what we know from the literature and the setup from Section 2.4, even before any simulation results.

When it comes to communicative success, types with $L_{\mathrm{bound}}$ have a functional advantage over $L_{\mathrm{lack}}$; literal $L_{\mathrm{bound}}$ has a slight advantage over pragmatic $L_{\mathrm{bound}}$; all have a sizable advantage over literal $L_{\mathrm{lack}}$. The red arrows in Fig. 1 sketch trajectories we can accordingly expect if there is only pressure for communicative success. How much each replicator step moves a population through this space ultimately depends on the rationality parameter *λ*. Functional differences decrease as *λ* increases: If *λ* is sufficiently high, all types except for literal $L_{\mathrm{lack}}$ speakers will show a strong tendency to associate $s_{\exists \neg \forall }$ with $m_{\mathrm{some}}$ and $s_{\forall }$ with $m_{\mathrm{all}}$.

Under probability matching, *l* = 1, iterated Bayesian learning converges to the prior over types (Griffiths & Kalish, 2007). If the prior on types is uniform, populations gravitate toward the space’s center under repeated mutator steps. This is sketched by the blue dash‐dotted arrows in Fig. 1. If instead the prior favors $L_{\mathrm{lack}}$ over $L_{\mathrm{bound}}$, we expect repeated mutator steps to drive the population toward the upper half of the space. This is suggested by the solid purple arrows in Fig. 1. The system’s predictions are more difficult to predict when *l* > 1, because now individual mutator steps may also be influenced not only by the prior but also by the differential likelihood with which a particular type *i* can be confused for any other type *j* (see Section 2.4.3 and Appendix B).

Despite its simplicity, the diagram in Fig. 1 makes clear the sense in which semantics and pragmatics might coevolve. Pragmatic language use will not evolve under strong and fully expressive semantics (bottom part of Fig. 1). A weak semantic convention will not evolve without disposition toward pragmatic enrichments (left part of Fig. 1). But together a weak semantics and a pragmatic disposition for enrichment can coevolve. These abstract theoretical considerations also suggest that we need both fitness‐based selection of communicative efficiency and pressure toward learnability to see underspecified semantics and pragmatic language use emerge together.

**Figure 2:**
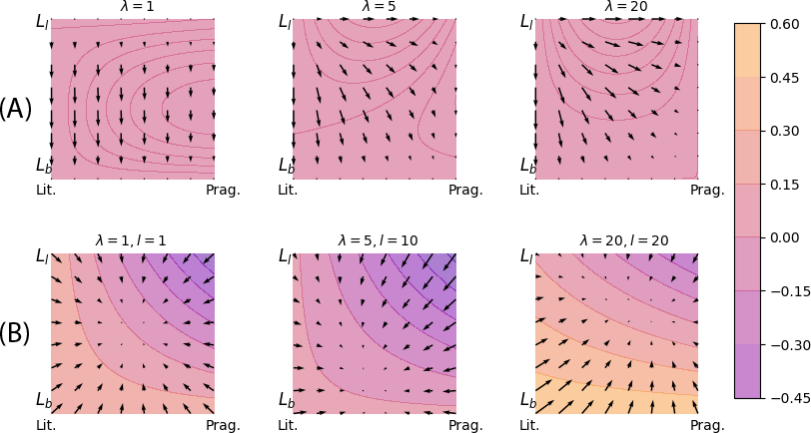
Dynamics on a two‐dimensional type space with four types. The *y*‐axis represents the proportion of types with $L_{\mathrm{lack}}$; the *x*‐axis that of pragmatic ones. Arrows indicate directionality of trajectories after (a) replicator steps and (b) mutator steps with a prior that favors types with $L_{\mathrm{lack}}$ by a factor of 1.05 over those with $L_{\mathrm{bound}}$. Colored contours show the proportion of pragmatic $L_{\mathrm{lack}}$ users after a step.

Fig. 2 shows actual evolutionary trajectories in this type space. The replicator steps shown in the top row of Fig. 2 make the functional disadvantage of $L_{\mathrm{lack}}$ against $L_{\mathrm{bound}}$ apparent. With increasing *λ*, this difference is mitigated for pragmatic users of $L_{\mathrm{bound}}$, who are no longer selected against by fitness‐based selection.

As for the iterated learning only, the diagrams in the bottom row of Fig. 2 show the case of a prior which favors types with underspecified semantics ($L_{\mathrm{lack}}$) over those with lexicalized upper bounds ($L_{\mathrm{bound}}$) by a factor of 1.05. There are three main things to note. First, as expected from the literature, a small bias for a type will lead to larger proportions of types who adopt it under *l* = 1 (see lower left space in Fig. 2, where the system gravitates indeed to a population where the relative frequency of $L_{\mathrm{lack}}$ over $L_{\mathrm{bound}}$ is exactly 1.05). Second, if *l* > 1, the dynamic is influenced heavily also by asymmetries in production likelihoods. Third, these asymmetries in the production likelihoods are in turn influenced by *λ*, so that *λ* also has an effect on iterated Bayesian learning (compare the middle and right‐most diagram in the bottom row of Fig. 2).

Figs. 1 and 2 investigate replicator and mutator steps in isolation. It remains to investigate how exactly these forces shape a population over time when they apply together. This will also hinge on which types are present because the space of all types determines how much production likelihoods asymmetrically favor certain types over others (when *l* > 1; see Appendix B). For this reason, we turn to a somewhat more realistic but still manageable setup with a larger set of types next.

### 3.2 Setup

#### 3.2.1 States, messages, lexical representations, and lexica

Consider a state space with three states $S\;=\;\{\emptyset ,\;s_{\exists \neg \forall },\;s_{\forall }\}$ and think of it as a partition of possible worlds into cells where none, some, or all of the *A*s are *B*s, for some arbitrary fixed predicates *A* and *B*. Eight lexical representations can be distinguished based on their truth or falsity in three world states, six of which are not contradictory or tautological (see Table 2 below).

A lexicon *L* is a mapping *M*→*R* from messages to representations. With three messages there are $6^{3}\;=\;216$ possible lexica. Some assign the same representations to more than one message and others lexicalize the same representations but associate them with different messages. Out of these possible lexica, three kinds are of particular relevance. First, lexica that assign the same lexical representations to more than one message. Such lexica lack in expressivity but may be favored by particular learning biases nonetheless (see below). Second, lexica that conventionalize upper bounds to realize a one‐to‐one mapping of messages to states. Finally, lexica that do not lexicalize upper bounds but would allow for perfect communication under additional pragmatic strengthening. There are six lexica of the second kind and six of the third. The following three lexica exemplify each kind: 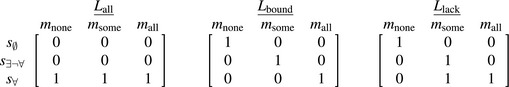


Recall that types are a combination of a lexicon and a manner of language use. We analyze the model’s predictions in populations of types with one of the two behaviors introduced earlier: literal or pragmatic. The former correspond to level‐0 reasoners and the latter to ones of level 1. Accordingly, there is a total of 432 types. Six types are pragmatic language users with $L_{\mathrm{lack}}$‐like lexica. We refer to these as *target types* because they represent lexica and language use that conform to the majority view of scalar implicatures. Twelve types are either literal or pragmatic users of lexica of the $L_{\mathrm{bound}}$ kind. We refer to these as *competitor types*, because they are expected to be the target types’ main contenders in evolutionary competition.

Note that while different types may lexicalize the same representations, they may nevertheless map different states to different overt messages. Consequently, different types of the same kind will fail to understand each other completely.

#### 3.2.2 An inductive learning bias for semantic simplicity

There is a growing effort to develop empirically testable representational languages that allow for the measure of semantic complexity. For instance, so‐called *languages of thought* (LOTs) have been put to test in various rational probabilistic models that show encouraging results (see, for example, Katz, Goodman, Kersting, Kemp, & Tenenbaum, 2008; Piantadosi et al. under review, 2012; and Piantadosi & Jacobs, 2016 for recent discussion). At its core, a LOT defines a set of operations and composition rules from which lexical representations can be derived. As a first approximation and for the sake of concreteness, we follow this approach to motivate and formalize a preference of learners for simpler semantic representations (Chater & Vitányi, 2003; Feldman, 2000; Kirby et al., 2015; Piantadosi, Tenenbaum, & Goodman, 2012; Piantadosi et al. under review). In a weighted generative LOT, a representation’s complexity is a function of its derivation cost.

Our toy grammar of lexical representations is given in Table 1. This grammar uses basic set‐theoretic operations to form expressions which can be evaluated as true or false in states *s*∅, *s*∃¬∀, or *s*∀ from above. Applications of generative rules have a cost attached to them. Here, we simply assume that the formation of Boolean combinations of representations incurs two cost units, while all other rule applications incur only one cost unit. Table 2 lists all six lexical representations relevant here, their truth conditions, and the simplest formula that expresses this representation in the grammar in Table 1.

**Table 1:** Toy grammar in a set‐theoretic LOT with weighted rules

$R\to_{2}R\wedge R$	$R\to_{2}\neg R$	
$R\to_{1}X\subseteq X$	$R\to_{1}X\neq \emptyset$	$R\to_{1}X=\emptyset$
$X\to_{1}\left\{A,B\right\}$	$X\to_{1}X\cap X$	$X\to_{1}X\cup X$

**Table 2:** Available lexical representations and their minimal derivation cost

Intuitive Name	$s_{\emptyset }$	*s*∃¬∀	*s*∀	Least Complex Formula	Complexity
“all”	0	0	1	*A* ⊆ *B*	3
“some but not all”	0	1	0	A∩B≠∅∧A≠∅	8
“some”	0	1	1	*A* ∩ *B*≠∅	4
“none”	1	0	0	*A* ∩ *B*∅	4
“none or all”	1	0	1	¬(A∩B≠∅∧A≠∅)	10
“not all”	1	1	0	¬(*A*⊆*B*)	5

The complexity measures for lexical representations from Table 2 are used to define a learning bias that favors simpler representations over more complex ones. The prior probability of a type is just the prior probability of its lexicon. The prior of a lexicon is a function of the complexity of the lexical representations in its image set. Lexica with simpler representations accordingly have a higher prior. One simple way of defining such priors over lexica (and thereby types) is:

$$P\left(L\right)\propto \prod_{r\in Im(L)}\;P\left(r\right),\;\text{with}\;P\left(r\right)\propto \max_{r^{\prime }}Compl\left(r^{\prime }\right)-Compl\left(r\right)+1,$$

where *Compl*(*r*) is the complexity of the minimal derivation cost of representation *r* according to the LOT‐grammar (see Table 2). Applied to our space of lexica, this construal assigns the highest probability to a lexicon of type $L_{\mathrm{all}}$, which only uses the simplest lexical representation “all” for all messages. Lexica of type $L_{\mathrm{lack}}$ are less likely, but more likely than $L_{\mathrm{bound}}$.

The menu of inductive biases argued to shape language acquisition is steadily being refined. Apart from simplicity, prominent examples include mutual exclusivity (Clark, 2009; Merriman & Bowman, 1989), regularization (Hudson, Kam, & Newport, 2005), and generalization (Smith, 2011). Even when these biases are considered in isolation, there are many ways in which they can be translated into priors over types. The key assumption here, common to all simplicity‐biased priors, is that simple representational expressions should be favored over more complex ones (see, for example, Goodman, Tenenbaum, Feldman, & Griffiths, 2008; Kirby et al., 2015; Piantadosi et al., 2012). In view of these numerous possibilities, we should stress that these details—from the generative grammar to its complexity measure—are to be regarded as one convenient operationalization of one general approach to explicating learning biases; this is not an implicit commitment to the claim that this particular instrumentalization is the single most plausible. We merely want to have a maximally concrete working example in which the priors over types are systematically related to the complexity of lexical representations. We return to this issue in Section 4.

### 3.3 Simulation results

Recall that there are three parameters held constant across types: soft‐max parameter *λ* affects how strongly speakers favor messages that appear best from their subjective point of view; the bottleneck size *k* influences how faithfully learners can identify their teacher type; *l* defines the learners’ disposition toward choosing the most likely teacher type from the posterior distribution. Based on considerations and results from Section 3.1, we expect that competitor types (types with lexica of the kind $L_{\mathrm{bound}}$) have a fitness advantage over target types (pragmatic types with lexica of the kind $L_{\mathrm{lack}}$), especially for very low levels of *λ* (see Fig. 1). Selection based on fitness alone may, therefore, not lead to prevalence of target types in the population. On the other hand, lexica of type $L_{\mathrm{lack}}$ are simpler than those of type $L_{\mathrm{bound}}$ by the postulated measure from above. This may make them more likely to be adopted by learners, especially when *l* is high. Still, lexica of the kind $L_{\mathrm{all}}$ are in turn even more likely a priori than lexica of the kind $L_{\mathrm{lack}}$. Simulation results will shed light on the question whether target types can emerge, and for which parameter constellations this is likely.

As before, we first look at the behavior of the replicator and mutator step in isolation, and then in combination. All simulation runs are initialized with an arbitrary distribution over types, constituting a population’s first generation. All reported results are the outcome of 50 update steps. These outcomes correspond to developmental plateaus in which change is, if not absent, then at least very slow. In other words, even if the resulting states do not correspond to an eventual attracting state, they characterize population states in which the system remains for a long time. As specified in Section 2.4.3, the mutation matrix *Q* can be obtained by considering all possible state‐message sequences of length *k*. Given that this is intractable for large *k*, the sets of data which learners are exposed to are approximated by sampling 250 *k*‐length sequences from each type’s production probabilities.

#### 3.3.1 Replication only: Selection based on communicative success

**Figure 3:**
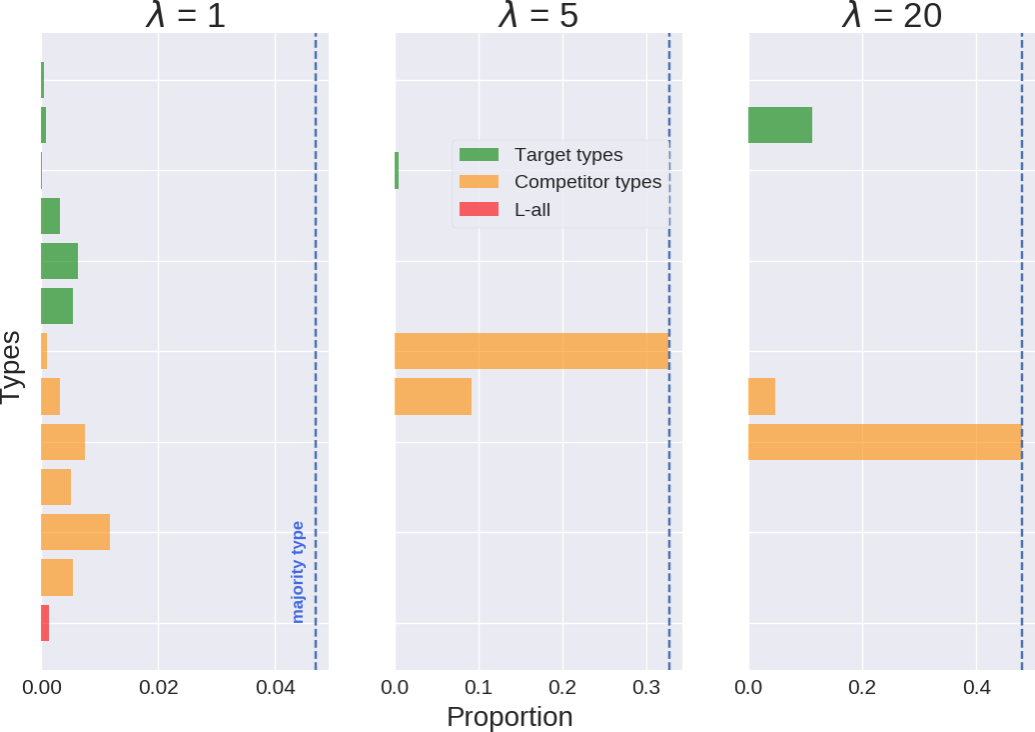
Proportion of target types, the six competitor types with the highest proportion, the most frequent type of the $L_{\mathrm{all}}$‐kind, and the population’s majority type, in representative populations after 50 generations under only a pressure for communicative success (replicator steps).

Selection based on communicative success is sensitive to *λ* since *λ* influences signaling behavior, which in turn determines communicative success. Fig. 3 shows the proportion of target types, the highest competitor types, and the highest type with an $L_{\mathrm{all}}$‐style lexicon in a representative population after 50 replicator steps. The plot also indicates the proportion of the *majority type*: the type with the highest proportion in the population. With low *λ* many types have very similar behavior, so that evolutionary selection lacks grip and becomes very slow. The result is a very long transition with near stagnancy in a rather homogeneous population with many types. Conversely, higher *λ* promotes less stochastic linguistic behavior, widening the gap in expressivity between some types and promoting more homogeneous populations. As suggested by Fig. 3, under replication only the majority in most populations is not one of the six pragmatic $L_{\mathrm{lack}}$‐style types. That is, a pressure only for communicative success does not lead to a prevalence of target types under any *λ*‐value. For instance, with *λ* = 20, 1,000 independent populations only had 11 cases in which a target type was the majority type, corresponding to a mean proportion of .003 across populations. In contrast, in 913 cases the majority types had $L_{\mathrm{bound}}$ with close to an even share between literal (454) and pragmatic types (459), corresponding to a mean proportion of about .48 taken together. In sum, fitness‐based selection of single types requires sufficiently high *λ* but does not often single out types with an underspecified semantic representation.

#### 3.3.2 Iterated learning only: Transmission fidelity and learnability

**Figure 4:**
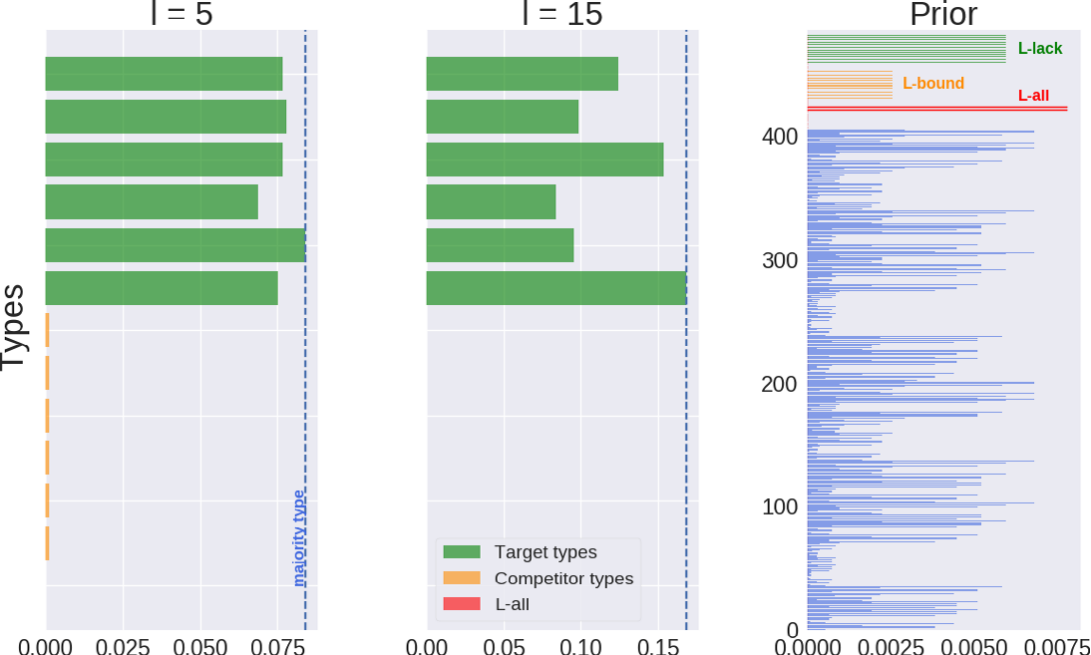
Proportion of target types, the six competitor types with the highest proportion, the highest type of the $L_{\mathrm{all}}$‐kind, and the population’s majority type in representative populations after 50 generations under only a pressure for learnability (*λ* = 20, *k* = 5). The learning prior is shown in the right‐most plot, with top‐most groupings corresponding to types, literal and pragmatic, with lexica of kinds $L_{\mathrm{lack}}$, $L_{\mathrm{bound}}$, and $L_{\mathrm{all}}$.

Recall that iterated learning without pressure for communicative success and posterior sampling (*l* = 1) converges to a population that mirrors the prior (Griffiths & Kalish, 2007), shown in the left‐most pane of Fig. 4. Its effects when learners exhibit a stronger tendency toward posterior maximization are illustrated in the other two plots of Fig. 4. The prior shows that while users of $L_{\mathrm{lack}}$ are not the most favored by the inductive bias (compared, for example, to $L_{\mathrm{all}}$), they are nevertheless more advantaged than others, such as $L_{\mathrm{bound}}$, in virtue of the relatively simple semantics they conventionalize. In contrast to, for example, $L_{\mathrm{all}}$, however, $L_{\mathrm{lack}}$ enables its users to convey each state with a single message when combined with pragmatic reasoning and sufficiently high *λ*. This makes it less likely to be confused with other types if the learning data are not too sparse (*k* ≥ 5) and *λ* not too low (*λ* ≥ 5). What is more, learners have a propensity to infer pragmatic $L_{\mathrm{lack}}$ when the teacher’s type produces very similar data, such as when using $L_{\mathrm{bound}}$ (see Section 3.1). As a consequence, a stronger propensity to maximize the posterior increases their proportion in the population. In 1,000 independent populations with *λ* = 20 and *l* = 5, all majority types were target types, with each reaching approximately the same proportion of users in the population.

Crucially, in contrast to a pressure only for communicative success with high *λ* (see Fig. 3), learnability alone does not succeed in selecting for a single prevalent type; all six target types tend to coexist at roughly equal proportion. Each is passed on to the next generation with the same faithfulness and, differently from a pressure for communicative success, they do not stand in competition with each other.

As with a pressure only for communicative success, low values of *λ* make the differences in observable behavior across types less pronounced. This makes differences in the likelihood of particular types having generated a learning input less pronounced. Therefore, low *λ* leads populations to reflect the learners’ inductive bias more faithfully. This favors functionally deficient but a priori preferred types such as those that use $L_{\mathrm{all}}$. A pressure for learnability alone may consequently lead to a spread of communicatively suboptimal types that lexicalize simpler semantics. For higher *λ* and at least a slight tendency to maximize the posterior, it becomes clear that a high prior is not the only thing that counts when it comes to learnability (see Section 3.1 and Appendix B). As soon as there is information for learners to discern whether one type is more likely to have generated the data (depending on *λ* and *k*), it becomes paramount for types to produce data that make them easily identifiable if they are to be inferred more often.

In sum, when pressured for learnability, pragmatic $L_{\mathrm{lack}}$ is promoted over functionally similar but semantically more complex alternatives such as $L_{\mathrm{bound}}$. However, learnability alone does not foment the propagation of a single target type across the population, because it does not differentiate between different ways of mapping the same semantic representations onto different overt signals.

#### 3.3.3 Combining pressures of communicative success and learnability

**Figure 5:**
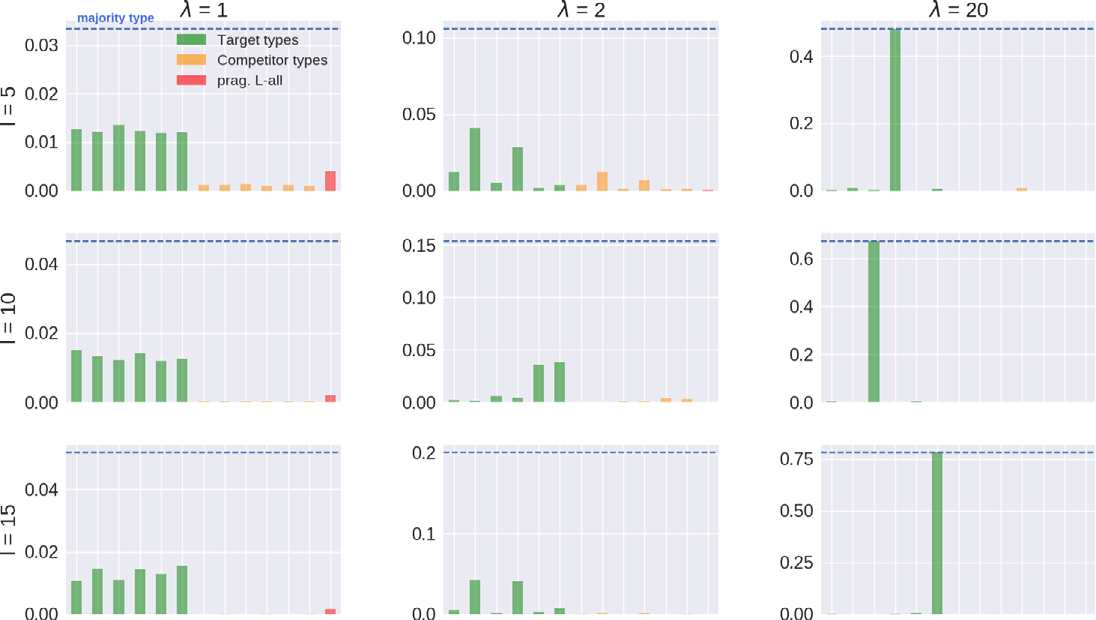
Proportion of target types, the six competitor types with the highest proportion, the highest type of the $L_{\mathrm{all}}$‐kind, and the population’s majority type in representative populations after 50 generations under both pressures (*k* = 5).

Pressure for communicative success and learnability is not sufficient on their own to have a single target type dominate the population. But the combination of both pressures can lead to the selection of a single target type (see Fig. 5). The proportion of a single majority target type increases with *λ* and *l*.

**Figure 6:**
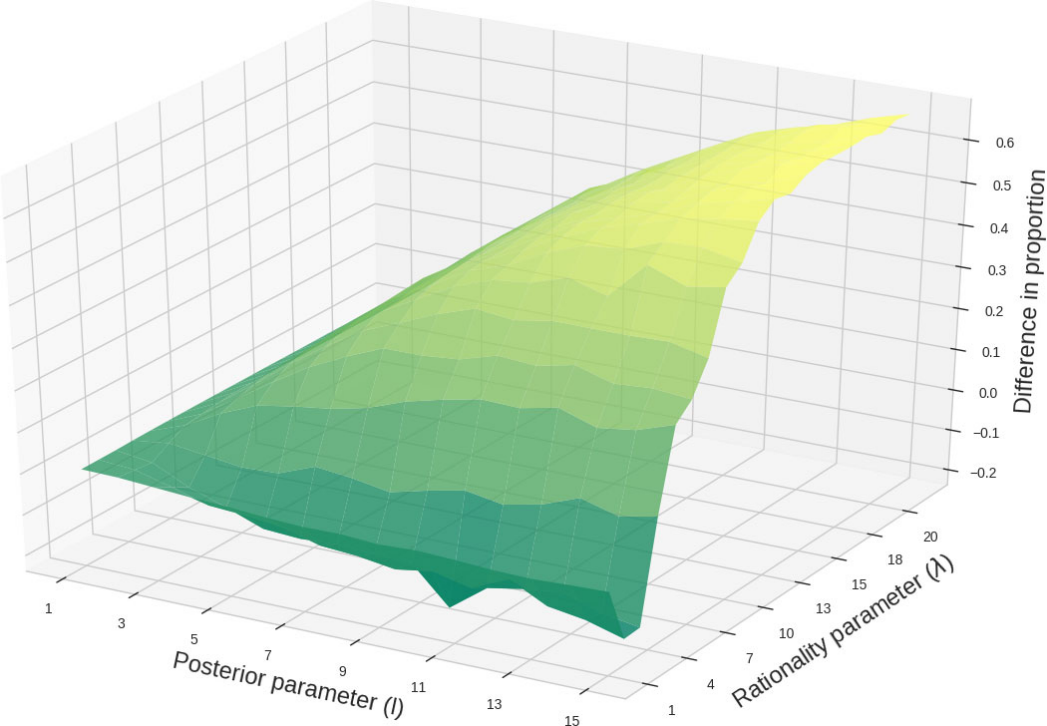
Mean difference between proportion of highest target type and highest other type in 1,000 independent populations after 50 generations under both pressures (*k* = 5).

As before, low *λ* and *l* lead to the prevalence of communicatively suboptimal types that are a priori favored, such as $L_{\mathrm{all}}$. An increase in *λ* leads to the selection of target types but does not necessarily lead to monomorphic populations if learners’ tendency to maximize the posterior is not very strong (see the uppermost row in Fig. 5) or absent. Finally, a combination of high *λ* and *l* leads to increasing proportions of a single majority target type. This joint influence is summarized in Fig. 6, which shows the mean difference between the most frequent target type and the proportion of the most frequent nontarget type in 1,000 independent populations across *λ* and *l* values. Higher values of *λ* and *l* increase the prevalence of a single target type, whereas lower values lead to less pronounced differences, with a valley resulting from low *λ* and high *l* (cf. distance to the majority type in Fig. 5 for *λ* = 1).

Effects of manipulating the sequence length *k* have not been addressed so far, but are rather predictable: Small values lead to more heterogeneous populations that reflect the learner’s prior more faithfully. This is due to the fact that the likelihood that a small sequence was produced by any type is relatively uniform. In contrast, larger values increasingly allow learners to differentiate types with different signaling behaviors.

In sum, when pressured for communicative success only, target types are outperformed by competitor types. When pressured for learnability only, populations are polymorphic due to lack of competition. When we combine both pressures, the (slight) functional disadvantage of targets is counterbalanced by an advantage in learnability. This leads to large proportions of targets and, due to the competition among types applied by each replicator steps, to more monomorphic populations. To conclude, target types can come to dominate the population if three assumptions are met: (i) language is pressured toward both communicative success *and* learnability; (ii) pragmatic language use is an option; (iii) learners prefer simpler over more complex lexical representations and exhibit a tendency toward the acquisition of the type that best explains the learning data.

## 4 General discussion

The approach introduced here combines game‐theoretic models of functional pressure toward successful communication (Nowak & Krakauer, 1999), effects of transmission perturbations on (iterated) language learning (Griffiths & Kalish, 2007), probabilistic speaker and listener types of varied degrees of pragmatic sophistication (Frank & Goodman, 2012; Franke & Jäger, 2014), as well as reasoning about unobservable lexical representations (Bergen, Goodman, & Levy, 2012; Bergen, Levy, & Goodman, 2016). This allows for a conceptual investigation of the (co‐)evolution of conventional meaning and pragmatic language use. Main contributions of the model are (i) its modular separation of communicative success and learnability on evolutionary trajectories, (ii) the characterization of language learning as a joint inference over linguistic behavior and lexical meaning, and (iii) the possibility to trace the coevolution of conventional semantics and pragmatic use.

With respect to (i), Kirby et al. (2015) propose a comparable model of the interaction between a lexicon’s expressivity and its learnability. A main difference is that here we considered communicative pressure for mutual understanding. This pressure can indirectly select for expressive types, those that can convey states unequivocally, whereas Kirby et al. only consider the bearing that the latter ability has on the production of learnable data. We see three main reasons for considering communicative success rather than just expressivity, and for looking at communication and learning rather than just learning. First, learning alone can promote populations with non‐negligible proportions of functionally defective types. This is true both of simulations and of laboratory experiments with human subjects (see Kirby, Cornish, & Smith, 2008; Silvey, Kirby, & Smith, 2014; see Fay & Ellison, 2013 for review of laboratory results). Second and more important, types may be equally expressive but their performance as a means of information transfer depends not only on themselves but on the population they find themselves in (compare the competition of target types in Fig. 5 and their lack of competition in Fig. 4). That is, we contend that the adoption and retention rate of an expressive type that generates learnable data does not, in itself, capture a type’s arguably central communicative function of transferring information to peers. Taking communication into consideration allows the model to be responsive to the task for which language is learned. Lastly, chains of iterated learning alone do not put types in direct competition. Accordingly, learning alone leads to polymorphous populations in which multiple types of a kind coexist (Nowak, 2006).

The main result of our case study is that types that correspond to the majority view of scalar implicatures—scalar readings are nonlexicalized pragmatic enrichments—can come to dominate a population. This can happen if pragmatic language use is recruited indirectly by a preference for simpler lexical representations (relative to more complex ones that lead to comparable overt linguistic behavior without pragmatic language use). Under this view, semantics and pragmatics play a synergic role and can coevolve: Pragmatic use allows maintenance of simpler representations; pressure toward representational simplicity indirectly promotes pragmatic over literal language use.

While the results of this case study are interesting, they also raise a number of critical issues. First of all, while many favorable parameter settings exist which lead to a prevalence of target types, other types are usually represented in non‐negligible proportions as well (see Fig. 5 and 6). This may just be a technical quirk of the mutator step; but there is a related issue of empirical importance. Several experimental studies on scalar implicatures suggest that participants can be classified as either semantic or pragmatic users of, in particular, *some* (e.g., Bott & Noveck, 2004; Degen & Tanenhaus, 2015; Nieuwland, Ditman, & Kuperberg, 2010). The former consistently accept *some* where *all* would be true as well, the latter do not. Interestingly, in our simulations, when a target type is the majority type, an inflated proportion of the population uses compatible lexica with a lexicalized upper bound. In other words, we find a tendency toward a similar coexistence of semantic and pragmatic types. Whether this analogy has any further explanatory value is an interesting path for future exploration.

Another important issue that is not addressed in the present model are potential costs associated with pragmatic reasoning. Here, we simply assumed that literal and pragmatic reasoning strategies exist from the start and are equally costly to apply. In contrast, empirical results suggest that the computation of a scalar implicature may involve additional cognitive effort (e.g., Breheny, Katsos, & Williams, 2006; Huang & Snedeker, 2009; Neys & Schaeken, 2007; Tomlinson, Bailey, & Bott, 2013). Extensions of the model presented here to include processing costs for pragmatic language use would be interesting future work. It seems plausible that effects of reasoning cost may tradeoff with the frequency with which a given scalar expression is used. It may be that frequently drawn scalar implicatures lexicalize to avoid cost, whereas infrequent ones are derived online to avoid more complex lexical representations during acquisition. Such a prediction would lend itself to empirical testing in line with a recent interest in differences between various scalar implicature triggers (van Tiel, van Miltenburg, Zevakhina, & Geurts, 2016).

We tried to motivate and formalize a general assumption about lexical representations’ complexity with a concrete, albeit provisional proposal. The specification of a learning bias in terms of a “grammar of representations” can and should be seen critically, however. Much depends on the primitives of such a grammar. For instance, the lexical representation “none or all” is the most complex in Table 2. But consider adding a new primitive relation between sets *A*⌣*B* which is true if and only if ¬(A∩B≠∅∧A≠∅). The lexical representation “none or all” would then be one of the simplest. Clearly, further research, empirical and conceptual, into the role of representational complexity, processing costs, and learning biases is needed. The model here makes a clear and important contribution nonetheless: It demonstrates how simplicity of representations can interact with use and evolutionary selection and shows that for simple representations to emerge it may require pragmatic strategies to compensate their potential expressive deficiencies. Hence, a model of coevolving semantics and pragmatics is needed. Future work should also include the possibility that representational simplicity may itself be a notion that is subject to evolutionary pressure (cf. Thompson et al., 2016), as well for the evolution of elements that define the agents’ cognitive make‐up: *λ* and *l*.

This case study is a first attempt at an explanation of how scalar implicatures evolved. But other factors, which are presently not taken into account, should be considered eventually even if they will lead to much more complex modeling. One such factor is the observation that nonlexicalized upper bounds allow a broader range of applicability, for example, when the speaker is not certain as to whether *all* is true. This may suggest an alternative and purely functionalist argument for why upper bounded meanings do not conventionalize: Should contextual cues provide enough information to the hearer to identify whether a bound is intended to be conveyed pragmatically, then this is preferred over expressing it overtly through longer expressions, for example, by saying *some but not all* explicitly. Importantly, although morphosyntactic disambiguation may be dispreferred due to its relative length and complexity (Piantadosi et al., 2012), it allows speakers to enforce an upper bound and override contextual cues that might otherwise mislead the hearer. In a nutshell, this explanation posits that scalar implicatures fail to lexicalize because, all else being equal, speakers prefer to communicate as economically as possible and pragmatic reasoning enables them to do so. What this alternative argument does not explain is why functional pressure does not lead to the emergence of different, equally costly lexical items to express different knowledge states of the speaker (Horn, 1972, 1984:252‐267; Traugott, 2004, van der Auwera, 2010). That is, this argument does not explain why English and other languages do not have a monomorphemic dual for, for example, *some* that lexicalizes an upper bound. If this hypothetical expression existed, it could be deployed to signal that the speaker knows that *some but not all* holds, and unbounded *some* could exclusively signal epistemic uncertainty. Looking at pressure from learnability might come in again.

Beyond scalar implicatures, the model can generate predictions about likely lexicalization trajectories of pragmatic inferences, or a lack thereof. In this realm, an interesting issue is whether proposed principles, such as the semantic conventionalization of once highly context‐dependent inferences, if they become regular enough (Levinson, 2000; Traugott, 2004), can be given a formal rationale and inform postulated directionalities of change. The present investigation made a first start and gave a framework for exploring these issues systematically.

## 5 Conclusion

The cultural evolution of meaning is influenced by intertwined pressures. We set out to investigate this process by putting forward a model that combines pressure toward successful information transfer with perturbations that may arise in the transmission of linguistic knowledge in acquisition. Its objects of selection and replication are pairs of lexical meanings and patterns of language use. This allows the model to trace the evolutionary interaction between conventional meaning and pragmatic use. Additionally, it takes the challenge seriously of neither semantics nor pragmatics being directly observable. Instead, learners need to infer these unobservables from overt data that result from their combination. These components and their mutual influence were highlighted in a case study on the lack of lexical upper bounds in weak scalar expression. This study showed that, when pressured for learnability and communicative success, the former force can drive for simpler semantic representations inasmuch as pragmatics can compensate for lack of expressivity in use. That is, the relative learning advantage of simpler semantics in combination with functional pressure in use may offer an answer to why natural languages fail to lexicalize systematic pragmatic inferences. Conversely, by appealing to the economy of lexical representations, this model also suggests a rationale for why a system of pragmatic enrichment could have evolved in the first place. The resulting picture is one of coevolution: The division of labor between semantics and pragmatics could have evolved because underspecified lexical meanings are easier, yet can only be maintained with a mechanism of pragmatic enrichment; pragmatic reasoning, however, would not evolve in the absence of lexically ambiguous representations.

## 6 A minimal example of the replicator mutator dynamic

Consider a simple and abstract coordination game. Agents are of two types: positive or negative. If agents of different types interact with each other, they obtain a payoff of 0. If negative meets negative, each receives a payoff of 1. If positive meets positive, they get a payoff of 2. A population state is completely characterized by the proportion *x* of negatives. The fitness of negatives in population state *x* is $f_{n}\left(x\right)\;=\;x$, that of positives is $f_{p}\left(x\right)\;=\;2-2x$. The average fitness is $\Phi \left(x\right)\;=\;xf_{n}\left(x\right)+\left(1-x\right)f_{p}\left(x\right)\;=\;3x^{2}-4x+2$. Without mutation, the replicator dynamic will update *x* to $\text{RD}\left(x\right)\;=\;f(x)x/\Phi (x)\;=\;x^{2}/\Phi (x)$.

**Figure 7:**
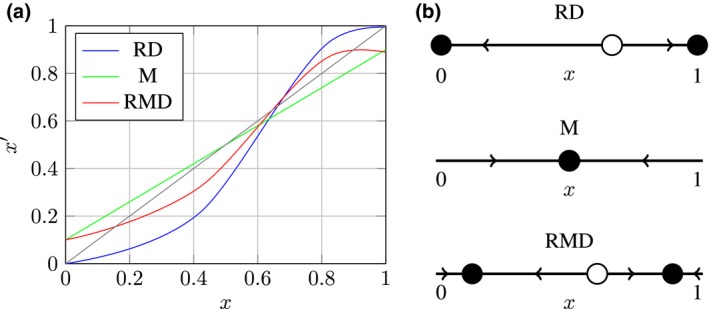
Example. (a) Update functions: the population state *x* is mapped onto $x^{\prime }$ in one update step. (b) Phase portraits for RD, M, and RMD: unstable rest points are hollow, and attractors are solid.

The update function RD(*x*) of the replicator step is plotted in Fig. 7 as the blue line. Rest points, for which RD(*x*) = *x*, are at *x* = 0, *x* = 1 and *x* = 2/3. The former are attractors; nearby points converge to them. Points near *x* = 2/3 also move toward 0 or 1. This is schematically pictured in the topmost phase portrait in Fig. 7. Adding mutation changes the dynamic and its rest points. Let us assume that $Q_{\mathrm{ji}}\;=\;.9$ when *j* = *i*. This is the proportion of types that are replicated faithfully. Conversely, a proportion of .1 will change their type from positive to negative, and vice‐versa. The update effect of mutation on its own are described by M(*x*) = .9*x*+.1(1−*x*) = .8*x*+.1, plotted as the linear green line in Fig. 7. As shown in Fig. 7, in this example mutation alone has only one stable rest point. It is located at *x* = .5. If we first take the replicator step and then the mutation step in sequence, we obtain the replicator mutator dynamic $\text{RMD}\left(x\right)\;=\;M\left(\text{RD}\left(x\right)\right)\;=\;.9x^{2}-.2x+.2/3x^{2}-4x+2$, which is plotted in red in Fig. 7. The rest points are at *x* = .121, *x* = .903 and *x* = .609. The former two are attractors (see Fig. 7).

## Supporting information

Data S1. Deriving the replicator mutator dynamic from conditional imitation.

## Notes

1 A crucial assumption in all of the derivations of the replicator dynamic as a biological or cultural process of evolution is that agents themselves are rather limited in their strategic capacities. They are, for example, not able to innovate a strategy, that is, to just choose a type that is optimal given the current population state. They are also not able to strategically adapt their behavior to any particular agent they are interacting with on some given occasion. Such minimalism about the strategic capacities of agents is a conservative methodological choice. It is one thing to be able to demonstrate that phenomenon *X* is what a rational agent would do. This is useful but may not always be the most satisfactory answer to the question how *X* happened to evolve. It is another thing to show that even if no agent was individually capable of seeing *X* as the rational thing to do, *X* would still evolve if every agent at least sometimes, locally and myopically, tried to optimize its behavior, for example, by imitating what seems to be working well for others (Skyrms, 2010).

2 An alternative choice for the speaker’s utility function, frequently made in Bayesian approaches after Frank and Goodman (2012), is $\log H_{n}\left(s|m;L\right)$. Conceptually, the difference is that without the logarithm, utilities are derived from action choices, while with the logarithmic, utilities are derived from how closely the listener’s beliefs (after hearing an utterance) align with the speaker’s, as measure by Kullback–Leibler divergence. In an evolutionary context, where fitness comes from actual interactions with the environment, not just solemn beliefs held about it, the formulation without the logarithm may be slightly more appealing. Moreover, log‐based speaker utilities cause the speaker never to use false utterances. Since we aim here for a situation in which any type has a reasonable chance to be mistaken for any other based on their production behavior (see Section 2.4.3), we want speaker types which send any message in any state with positive probability.

3 The way the posterior over types is formed and how learners employ it are important design choices. There are other alternatives as well. For instance, instead of adopting a single type, learners could keep their uncertainty over types and base their production behavior on the posterior over types they form (Burkett & Griffiths, 2010). A straightforward operationalization of this idea would then see production defined as a two‐step process wherein a speaker first samples from her posterior and then produces utterances based on the type she sampled, as done in Kirby et al. (2015). Beyond adding complexity to the model, a disadvantage of this approach is that it could happen that a speaker that first uttered a Hungarian utterance speaks Quechua in the next, so to speak. We, therefore, maintain the simpler assumption of a single type being acquired in the present context, and we postpone further investigation of more complex type‐adoption schemes to future research.

4 This two‐dimensional representation underspecifies the exact proportions of types found in a mixed population. The space’s center, for instance, could be composed in equal parts of pragmatic $L_{\mathrm{lack}}$ and of literal $L_{\mathrm{bound}}$. But it could equally well be a population that has all four types in equal proportions. This level of granularity will nevertheless be sufficient for our purposes, as we are only interested in sketching rough trajectories after replicator or mutator steps.

## Appendix A Example of iterated Bayesian learning

### A.1

We consider three types and three possible data observations they can produce. Types are equally likely a priori but show different and crucially asymmetric production behavior. The likelihood with which type $t_{i}$ produces datum $d_{j}$ is $LH_{\mathrm{ij}}$:If δ > *ε*, production likelihoods are asymmetrically skewed to favor $t_{1}$, in the sense that both types $t_{2}$ and $t_{3}$ produce behavior that is strong evidence for $t_{1}$ more often than behavior that is strong evidence for any other type different from themselves. Type $t_{1}$ makes no such “asymmetric mistakes” and is more faithfully recognized based on its production behavior than the other types.

Using the definitions from Section 2.4.3, we calculate mutation matrices *Q* for *l* = 1 and *l* = 10 when *ε* = 0.1 and δ = 0.2:Despite flat priors over types, the mutation matrix for *l* = 10 is skewed toward $t_{1}$ in the sense that the probability of any type mutating to $t_{1}$, $\sum_{i}Q_{i1}\;=\;1.2$, is higher than the probability of $\sum_{i}Q_{\mathrm{ij}}\;=\;0.8$ for *j* ∈ {2,3}. For *l* = 1, these probabilities are all equal for all three types. Consequently, under iterated Bayesian learning with *l* = 1 the population will eventually converge to a uniform distribution over all three types in the limit, but for *l* = 10 it converges to a skewed population vector $\vec{x}\approx \left\langle 0.5,0.25,0.25\right\rangle$, where $t_{1}$ is a clear majority type. This demonstrates how iterated Bayesian learning with *l* > 1 can be sensitive to the set of types represented in the model, in particular when production likelihoods are asymmetrically in favor of some types. With probability matching learners (*l* = 1), these considerations do not play a role, as the system will gravitate to the prior distribution over types nonetheless.
