# Supplementary material for: Coevolution of Lexical Meaning and Pragmatic Use
Source: Cogn Sci. 2018 Oct 7;42(8):2757–89. doi: 10.1111/cogs.12681 (PMC6586156; doi:10.1111/cogs.12681)
Supplement: Supplementary file 1 — Data S1. Deriving the replicator mutator dynamic from conditional imitation. [file COGS-42-2757-s001.pdf]

Deriving the replicator mutator dynamic from conditional imitation

Consider a huge, virtually infinite population of agents. At any point in time each agent has a fixed type i which determines its fitness f_i at that moment in time when playing with an arbitrary member of the population. The relative frequency of type i is x_i with $\sum_i x_i = 1$. The average population fitness is $\Phi = \sum_k x_k f_k$. The mutation probability from j to i is Q_{ji}, with $\sum_k Q_{jk} = 1$. The proportion of type i at the next time step x'_i is given by the discrete-time replicator mutator dynamic:

$$x'_i = \sum_j \frac{x_j f_j Q_{ji}}{\Phi}\,, \tag{1}$$

We will derive this dynamic from an agent-level update scheme of conditional imitation. There are many variants on this derivation. Our choice is to be as simple as possible. Sandholm (2010) provides much more detail and discusses many further interesting derivations of similar evolutionary dynamics.

A simple conditional imitation dynamic runs as follows. Every now and then an agent is selected at random from the population and given a chance to possibly change its behavior. Call this agent (perhaps slightly misleadingly) *learner* L. The learner chooses a random agent from the population to possibly imitate. Call this agent (somewhat more misleadingly) *teacher* T. Whether L actually chooses to imitate the behavior of T depends on how good T's behavior is at the moment. For simplicity, we assume that L adopts T's type with probability f_j, where j is the type of T.[1] If L chooses to imitate T's behavior, it may not be able to directly observe T's actual type. Rather it may have to infer T's latent type from its overt behavior. The probability that L actually acquires type k when it chose to imitate a teacher of type j is Q_{jk}. In sum, we assume that learners have (at least roughly) an estimate of how good another agent's behavior is, decide to imitate that behavior conditionally on how good it is and the adopt whatever mechanism (type) they infer may cause the observed behavior.

This model of occasional conditional imitation gives rise to expected changes in relative frequencies in the population. Given a huge population, the expected probability $P(k \to i)$ that some agent of type k switches by conditional imitation to type i is the product of probabilities for the following four events:

1. a learner L of type k is sampled, which happens with probability x_k
2. a teacher T of type j is sampled, which happens with probability x_j
3. L chooses to imitate T, which happens with probability f_j

[1] We assume here that $f_i \in [0; 1]$ for all i. This is actually the case for the definition of fitness as communicative success from Section **??**. See Sandholm (2010) for more complex alternative conditional update rules that still derive the same population dynamic.

4. L infers T's type to be i, which happens with probability Q_{ji}

The average expected change $\dot{x}_i$ in proportion of type i is then:

$$\begin{aligned}
\dot{x}_i &= \sum_k P(k \to i) - \sum_k P(i \to k) \\
&= \sum_k x_k \sum_j x_j f_j Q_{ji} - \sum_k x_i \sum_j x_j f_j Q_{jk} \\
&= \sum_j x_j f_j Q_{ji} - x_i \sum_j x_j f_j \\
&= (x'_i - x_i)\Phi
\end{aligned}$$

In this way, the discrete-time replicator mutator equation in (1) captures the expected difference in relative frequency of type i from one discrete time step to the next (relative to current baseline fitness Φ, which is constant for all types, and so only governs the absolute speed of change).

References

Sandholm, W. H. (2010). *Population Games and Evolutionary Dynamics*. Cambridge, MA: MIT Press.
